# Multi-Instrumental Analysis Toward Exploring the Diabetic Foot Infection Microbiota

**DOI:** 10.1007/s00284-023-03384-z

**Published:** 2023-07-05

**Authors:** Michał Złoch, Ewelina Maślak, Wojciech Kupczyk, Paweł Pomastowski

**Affiliations:** 1 Centre for Modern Interdisciplinary Technologies, Nicolaus Copernicus University in Toruń, Wileńska 4 Str, 87-100 Toruń, Poland; 2 Chair of Environmental Chemistry and Bioanalytics, Faculty of Chemistry, Nicolaus Copernicus University in Toruń, Gagarina 7 Str, 87-100 Toruń, Poland; 3 Department of General, Gastroenterological and Oncological Surgery, Faculty of Medicine, Collegium Medicum, Nicolaus Copernicus University in Toruń, Gagarina 7, 87-100 Torun, Poland

## Abstract

The polymicrobial nature of diabetic foot infection (DFI) makes accurate identification of the DFI microbiota, including rapid detection of drug resistance, challenging. Therefore, the main objective of this study was to apply matrix-assisted laser desorption ionization time-of-flight mass spectrometry (MALDI TOF MS) technique accompanied by multiply culture conditions to determine the microbial patterns of DFIs, as well as to assess the occurrence of drug resistance among Gram-negative bacterial isolates considered a significant cause of the multidrug resistance spread. Furthermore, the results were compared with those obtained using molecular techniques (16S rDNA sequencing, multiplex PCR targeting drug resistance genes) and conventional antibiotic resistance detection methods (Etest strips). The applied MALDI-based method revealed that, by far, most of the infections were polymicrobial (97%) and involved many Gram-positive and -negative bacterial species—19 genera and 16 families in total, mostly *Enterobacteriaceae* (24.3%), *Staphylococcaceae* (20.7%), and *Enterococcaceae* (19.8%). MALDI drug-resistance assay was characterized by higher rate of extended-spectrum beta-lactamases (ESBLs) and carbapenemases producers compared to the reference methods (respectively 31% and 10% compared to 21% and 2%) and revealed that both the incidence of drug resistance and the species composition of DFI were dependent on the antibiotic therapy used. MALDI approach included antibiotic resistance assay and multiply culture conditions provides microbial identification at the level of DNA sequencing, allow isolation of both common (eg. *Enterococcus faecalis*) and rare (such as *Myroides odoratimimus*) bacterial species, and is effective in detecting antibiotic-resistance, especially those of particular interest—ESBLs and carbapenemases.

**Supplementary Information:**

The online version contains supplementary material available at 10.1007/s00284-023-03384-z.

## Introduction

Chronic wounds are one of the most devastating impairments related to diabetes among which diabetic foot infection (DFI) represents the most frequent and serious disorder [1]. Considering the growing number of diabetic (ca. 420 million patients so far) DFI is currently considered as a predominant trigger for lower extremity amputations worldwide [2]. Accurate deciphering of the infection causative agent determines taking effective treatment, however, the reliable identification of the DFI microbial patterns in diabetic patients is still challenging due to usually the polymicrobial nature of the infection [3]. Moreover, there is a growing need for reports on the DFI microbial compositions in specific geographical regions to provide local treatment guidelines [4].

Up to date, the DFI diagnosis mostly relies on the traditional culture method and phenotypic identification of the grown colonies or the application of molecular techniques, such as the 16S rDNA PCR amplification and sequencing [5]. While the former is time-consuming and presents limited identification accuracy, the latter is characterized by high sensitivity, discriminatory power, and allows skip the culturing step, however, it requires a well-equipped laboratory and highly qualified staff, which increases the costs of analysis [6]. More recently, matrix-assisted laser desorption ionization time-of-flight mass spectrometry (MALDI TOF MS) seems to be a good solution for mentioned limitations, since fast and cost-effective bacterial identification by MALDI TOF MS along with the multiplication of culture conditions provides high identification accuracy in relatively short time-to-results and opens the possibility for further investigation of biological features including antimicrobial susceptibility of isolates [7]. It was proved in the work Złoch et al. [8] where the accuracy of the reflected microbial patterns of the swab samples derived from DFI patients via MALDI technique significantly improved when the multiply culture conditions was applied.

Although clinicians should avoid antibiotic therapy that is unnecessary, nevertheless, successful treatment of DFIs calls for the administration of appropriate antibiotics [9]. Facing an increased rate of isolation of antibiotic-resistant pathogens, which can be observed in the past few decades, the selection of the effective antimicrobial therapy of DFI becomes more challenging than ever before [2]. In particular, this applies to the constantly growing number of multidrug-resistant (MDR) Gram-negative species such as extended-spectrum beta-lactamase (ESBL), carbapenemase-producing *Enterobacterales*, or MDR *Pseudomonas aeruginosa* [9]. The frequency of occurrence of MDR Gram-negative pathogens in different geographical area and treatment centers is widely variable, however, current data showing that besides developing countries, this problem is increasingly affecting European and other developed countries [2]. In view of this, reliable and fast detection of antibiotic-resistant Gram-negative pathogens becomes crucial for preventing the failure of the DFI treatment and further spreading of MDR [10].

Many different methods of detecting antibiotic resistance/susceptibility have found application in routine clinical practice. The most commonly used techniques are phenotypic tests such as disc diffusion or combined disc inhibitory tests, gradient minimal inhibitory concentration (MIC) strips (Etest), the Carba NP tests or most recently the modified carbapenem inactivation method (mCIM) [11, 12]. Despite their low costs and simplicity, their major drawbacks are the need for additional incubation which extends time-to-result and relative low specificity and selectivity [13]. Application of molecular methods such as targeted PCR assays including multiplex ones is more specific and selective as well as enables receive results faster (even < 4 h) and detect several different resistance genes in a single run [14, 15]. However, the use of the molecular approach is very often limited, especially in developing countries, due to high costs of the analysis, need for highly trained staff, or access to commercial databases and dedicated equipment [6].

In the last few years, special attention has been paid to the utilization of rapid biochemical assays based on antibiotic hydrolyzing activity detection, which opens the possibility of obtaining accurate and reliable results in a simple, fast, and cheap way [16]. In light of this, the MALDI TOF MS technique turned out to be the most promising tool facilitating the indication of a wide range of β-lactamases including clinically relevant cephalosporinases and carbapenemases [17]. Several publications have demonstrated the feasibility of the MALDI TOF MS technique [18–20]. Moreover, in the recent work Złoch et al. [21] authors pointed out that the MALDI could be also used to partially classify the class of the carbapenemase present in the sample or as a fast surrogate of standard MIC assay in case of metallo-β-lactamases producing strains (MBL).

The main goal of this study was to apply the MALDI microbial identification technique with the previously established multiple culture conditions to decipher microbial patterns of the swab samples collected from DFI patients treated in the Provincial Polyclinical Hospital in Toruń. (Poland). The accuracy of the MALDI TOF MS identification was evaluated by referring to the sequencing results of the 16S rDNA region. Moreover, as Gram-negative bacteria are considered the primary cause of the multidrug resistance spreading among DFI-suffering patients, the analysis of the frequency of the occurring resistance against different classes of beta-lactams, including cephalosporins and carbapenems using different approaches (MBT STAR BL assay, Etest, multiplex PCR) was performed for the sake of choosing the most accurate one.

## Materials and Methods

### Clinical Samples

During studies analyzed clinical specimens derived from 31 patients (42–85 yrs, 25 males, 6 females, Supplementary Table S1) of the Provincial Polyclinical Hospital in Toruń (Poland) who suffered from diabetic foot infections. The superficial samples of wounds were collected using flocked swab (ESwab Collection System, Copan) after wound debridement according to the local guidelines by a specialist nurse applying the Levine technique. The samples were immediately placed into a liquid transport medium (Amies δswab, Deltalab, Rubi Barcelona, Spain) and transported to *Centre for Modern Interdisciplinary Technologies (Nicolaus Copernicus University in Toruń),* where they were stored at − 80 °C.

### Bacteria Isolation and Culturing Technique

For bacteria isolation, serial dilution method (10^–1^–10^–3^) in sterile peptone water (Sigma Aldrich, Steinheim, Germany) was applied. After defrosting, samples were thoroughly vortexed and then 0.5 mL was transferred into the test tube containing 4.5 mL of sterile peptone water (Sigma Aldrich, Germany) and again vortexed (first dilution—10^–1^). 100 μL of each dilution was plated onto 5 different culture media previously selected as the most useful in DFI bacteria recovery [8]: Tryptic Soy Agar (TSA; Sigma Aldrich, Steinheim, Germany), Columbia Blood Agar (BLA; Oxoid, Basingstoke, Great Britain), CHROMagar Orientation (CHRA; GRASO Biotech, Starogard Gdański, Poland), Glucose Bromocresol Purple Agar (BCP; Sigma Aldrich, Steinheim, Germany), and Vancomycin Resistant Enterococci Agar (VRE; Oxoid, Basingstoke, Great Britain). All media were in the form of ready-to-use powders except for BLA, which was prepared by adding defibrinated sheep blood (GRASO Biotech, Starogard Gdański, Poland) to the sterilized and dissolved Colombia blood agar base to the final concentration 5% (v/v). Bacterial cultures were incubated at 37 °C for 24 h and then single colonies characterized by different morphological features were selected to obtain pure cultures using the streak plate method on the same media (incubation at 37 °C for 18–24 h).

### Identification of Bacterial Isolates Using MALDI TOF MS Technique

For bacteria identification used protein extracts obtained applying formic acid/acetonitrile method according to the MALDI Biotyper protocol (Bruker Daltonik GmbH, Bremen, Germany). 1 microbial loop (10 µl) of fresh biomass was suspended in 300 µl of sterile deionized water, mixed, and then viable bacterial cells were inactivated by adding 900 μL of 96% ethyl alcohol. After vortexing, the resulted bacterial suspension was centrifuged (1300 rev/min, 5 min), the supernatant was discarded and the remaining cell pellet was dried using a vacuum centrifuge at room temperature. Consequently, to the cell pellet 10 µl formic acid (FA) and 10 µl acetonitrile (ACN) was added and mixed. The obtained extract was centrifuged (13,000 rev/min, 5 min.) and 1 µl of supernatant was transferred onto a MALDI MTP 384 ground steel target sample spot (Bruker Daltonik GmbH, Germany). After air-drying, the sample spot was overlaid with 1 µl of MALDI matrix solution—10 mg/mL α-cyano-4-hydroxycinnamic acid (HCCA; Sigma Aldrich, Switzerland) solution prepared in standard solvent solution (50% ACN, 47.5% water and 2.5% trifluoroacetic acid).

Bacterial protein extracts were analyzed using an ultrafleXtreme MALDI TOF mass spectrometer (Bruker Daltonik GmbH, Bremen, Germany) equipped with the smartbeam-II laser–positive mode. Spectra were collected manually using manufacturer software, flexControl (parameters: 500 shots in-one-single spectra to frequency 2500, *m/z* range = 2000–20,000, acceleration voltage = 25 kV, global attenuator offset = 20% and attenuator offset = 34% and its range = 34%, laser power = 40%), and subjected to smoothing using the Savistsky-Golay method (width 2 m/z, cycles 10) and baseline corrections using the TopHat algorithm (signal to noise threshold 2; peak detection algorithm–centroid) followed by calibration using the Bruker's Bacterial Test Standard (BTS; Bruker Daltonik GmbH, Bremen, Germany) in quadratic mode via manufacturer software, flexAnalysis. Each sample was measured in quadruplicate (two spots per samples measured in twice). Validated mass spectra were used for bacterial identification via MALDI Biotyper 3.0 Platform (Bruker Daltonik GmbH, Bremen, Germany) based on the both raw spectra (RAW) and Main Spectra (MSP).

### Identification of Bacterial Isolates Using 16S rDNA Sequencing

First of all, the total bacterial genomic DNA were obtained using E.Z.N.A.® Bacterial DNA Kit (Omega Bio-tek, Norcross, US) from overnight bacterial cultures (37 °C) following extraction protocol with Glass Beads S supplied by the manufacturer. The extracted bacterial DNA were used for amplification of the 16S rDNA region via PCR technique using universal bacterial primers 27F (5-AGAGTTTGATCMTGGCTCAG-3) and 1492R (5-GGTTACCTTGTTACGACTT-3), thermostable *Taq* DNA polymerase (Qiagen, Hilden, Germany), Mastercycler pro S thermocycler (Eppendorf AG, Hamburg, Germany), and PCR program established in the earlier work [22]. The efficiency of the obtained PCR products and purity were studied quantitatively by spectrophotometry as well as gel electrophoresis in 1% agarose. Subsequently, PCR products were send to Genomed (Warsaw, Poland) and were sequenced via the Sanger dideoxy method using the same primers, contigs were assembled via BioEdit Sequences Alignment Editor ver. 7.2.5 [23], and consensus sequences were compared with references sequences in rRNA/ITS databases of the National Center for Biotechnology Information via the BLAST algorithm (https://blast.ncbi.nlm.nih.gov/Blast.cgi?PAGE_TYPE=BlastSearch). The DNA sequences determined in this study were submitted to GenBank, and accession numbers are given in the Table S1. The evolutionary tree of identified bacterial strain was inferred based on the Neighbor-Joining and Maximum Composite Likelihood method using MEGA7 software [24] and was visualized using Interactive Tree of Life (iTOL) v 6.5.4 [25].

### Determination of the Antibiotics Resistance Among Gram-Negative Strains Using Etest Method

Investigated Gram-negative strains were incubated on MHA medium (Mueller Hinton Agar; Sigma Aldrich, Steinheim, Germany) for 24 h at 37 °C. After incubation, bacterial suspensions at a density of 0.5 McFarland in saline (0.85%) were prepared. The suspension was applied to the plate with MHA medium using a swab soaked in it. One swab was used per plate. Then strips containing different antibiotic gradient (Etest®, Biomerieux) were applied: (1) ESBL CT/CTL 16/1—cefotaxime (0.25–16 μg/mL)/cefotaxime (0.016–1 μg/mL) + clavulanic acid (4 μg/mL); (2) ESBL TZ/TZL 32/4—ceftazidime (0.5–32 μg/mL)/ceftazidime (0.064–4 μg/mL) + clavulanic acid (4 μg/mL); (3) ESBL PM/PML 16/4—cefepime (0.25–16 μg/mL)/cefepime (0.064–4 μg/mL) + clavulanic acid (4 μg/mL); (4) ceftriaxone TX 32 (0.002–32 μg/mL); (5) MBL IP/IPI 256/64—imipenem (4–256 μg/mL)/imipenem (1–64 μg/mL) + EDTA; (6) ampicillin AM (0.016–256 μg/mL) as well as (7) ciperacillin PP (0.016–256 μg/mL). The MIC values were read from the zone of inhibition of bacterial growth after 20–24 h of incubation at 37 °C.

### Detection of Antibiotic Resistance Among Gram-Negative Isolates Using MALDI TOF MS Technique

Detection of β-lactamase activity against different beta-lactam antibiotics—cephalosporins (cefotaxime, ceftriaxone, ceftazidime), carbapenems (imipenem, meropenem) as well as penicillins (ampicillin, piperacillin) via MALDI TOF MS technique was performed using MBT STAR-BL method according to guidelines provided by manufacturer protocol (Bruker Daltonik GmbH, Bremen, Germany). One inoculation loop (1 µL) containing sufficient amount of bacterial cells (1 to 5 colonies) was suspended in the antibiotic solution: (1) cefotaxime (CTX, final conc. 0.5 mg/mL), (2) ceftriaxone (CRO, 0.5 mg/mL), (3) ceftazidime (CAZ, 0.5 mg/mL), (4) imipenem (IMI, 0.5 mg/mL), (5) meropenem (MER, 1.0 mg/mL), (6) ampicillin (AMP, 3.0 mg/mL), and (7) piperacillin (PIP, 2.0 mg/mL) dissolved in 50 µL of MBT STAR Buffer. Prepared mixtures were subjected to incubation at 35 ± 2 °C for 30 (CRO, IMI), 120 (CTX, MER, AMP), 180 (PIP) or 360 (CAZ) minutes according to manufacturer guidelines with constant shaking (900 rpm) using Thermomixer (Eppendorf AG, Germany). After incubation samples were centrifuged (2 min., 13,000 rpm) and 1 µL of supernatants were deposited onto the MALDI MTP 384 ground steel target (Bruker Daltonik GmbH, Bremen, Germany) in duplicate, air dried, and subsequently overlaid with 1 µL of MBT STAR Matrix. For each targets and runs prepared one spot with 1 µL of MBT STAR-ACS (antibiotic calibration mass standard containing dedicated masses below *m/z* = 1000) as well as both negative (*Escherichia coli* ATCC 25922) and positive (*Klebsiella pneumoniae* ATCC BAA-1705) controls in duplicate. Target plates were analyzed using ultrafleXtreme MALDI–TOF/TOF mass spectrometer (Bruker Daltonik GmbH, Bremen, Germany) equipped with the smartbeam-II laser–positive mode. The spectra were collected automatically via AutoXecute mode and flexControl software (Bruker Daltonik GmbH, Bremen, Germany) as well as calibrated using quadratic calibration method using flexAnalysis software (Bruker Daltonik GmbH, Bremen, Germany). Finally, 4 spectra for each sample were obtained. Collected spectra were analyzed by means of MBT Biotyper Prototype software and results were expressed as normalized logRQ values calculated from division signal intensity of hydrolyzed forms of antibiotic by those derived from non-hydrolyzed. For normalization of the results used the respective negative and positive controls. Higher logRQ means higher antibiotic hydrolysis. Normalized logRQ ≤ 0.2—negative results, ≥ 0.4—positive results, values between thresholds—unclear hydrolyzation.

### Detection of Antibiotic Resistance Genes Among Gram-Negative Isolates Using Multiplex PCR Technique

For detection of genes encoding different types of β-lactamases—ESBLs (TEM, SHV, CTX-M, OXA-1), carbapenemases (KPC, GES, OXA-48, GIM, NDM, VIM, IMP), and extended-spectrum AmpC (CMY-1, CMY-2)—used the same total bacterial genomic DNA isolated for 16S rDNA sequencing. Primer sets used for amplification of individual genes are listed in the Table 1. Amplification performed using thermostable *Taq* DNA polymerase (Qiagen, Hilden, Germany) and Mastercycler pro S thermocycler (Eppendorf AG, Hamburg, Germany). PCR conditions for individual set of primers selected according to references provided in the Supplementary Table S2. Products of amplification were analyzed using electrophoresis in a 1.5% agarose gel containing 0.05 mg/L ethidium bromide at 90 V for 1 h in 1 × TAE buffer (TAE buffer (50x) Molecular biology grad, AppliChem GmbH, Darmstadt, Germany). For determination of product size used Perfect™ 100 bp DNA ladder (100–2500 bp, EURx, Gdańsk, Poland) as well as DNA ladder MR19 (750–3500 bp, DNA Gdańsk, Blirt S.A., Gdańsk, Poland) (Table 2).

**Table 1:** List of primers used for amplification of individual genes encoding β-lactamases in single or multiplex PCR reaction

Set	β-lactamase	Primers	Sequence [5′ → 3′]	Product size [bp]	Ref.
1	TEM	MultiTSO-T.for	CATTTCCGTGTCGCCCTTATTC	800	[14]
		MultiTSO-T.rev	CGTTCATCCATAGTTGCCTGAC		
	SHV	MultiTSO-S.for	AGCCGCTTGAGCAAATTAAAC	713	
		MultiTSO-S.rev	ATCCCGCAGATAAATCACCAC		
	OXA-1	MultiTSO-O.for	GGCACCAGATTCAACTTTCAAG	564	
		MultiTSO-O.rev	GACCCCAAGTTTCCTGTAAGTG		
2	CTX-M-1	MultiCTXMGp1.for	TTAGGAArTGTGCCGCTGyA	688	
		MultiCTXMGp1-2.rev	CGATATCGTTGGTGGTrCCAT		
	CTX-M-9	MultiCTXMGp9.for	TCAAGCCTGCCGATCTGGT	561	
		MultiCTXMGp9.rev	TGATTCTCGCCGCTGAAG		
3	NDM	NDM-1-for	GGTTTGGCGATCTGGTTTTC	621	[15]
		NDM-1-rev	CGGAATGGCTCATCACGATC		
4	IMP	IMP-F	GGAATAGAGTGGCTTAAYTCTC	232	
		IMP-R	GGTTTAAYAAAACAACCACC		
	VIM	VIM-F	GATGGTGTTTGGTCGCATA	390	
		VIM-R	CGAATGCGCAGCACCAG		
5	GIM	GIM-F	TCGACACACCTTGGTCTGAA	477	
		GIM-R	AACTTCCAACTTTGCCATGC		
6	OXA-48	OXA-F	GCGTGGTTAAGGATGAACAC	438	
		OXA-R	CATCAAGTTCAACCCAACCG		
	KPC	KPC-Fm	CGTCTAGTTCTGCTGTCTTG	798	
		KPC-Rm	CTTGTCATCCTTGTTAGGCG		
7	GES	GES_For	CTGGCAGGGATCGCTCACTC	600	[26]
		GES_Rev	TTCCGATCAGCCACCTCTCA		
8	CMY-1	CMY-1_For	ATGCAACAACGACAATCCATCCTG	1560	[27]
		CMY-1_Rev	TCAACCGGCCAACTGCGCCAGGAT		
	CMY-2	CMY-2_For	ATGATGAAAAAATCGTTATGCT	3202	
		CMY-2_Rev	TTATTGCAGCTTTTCAAGAATGCG

**Table 2:** List of the DFI patients subjected to the studies

Patient	Gender	Age	Antibiotic therapy	Description
DFI-1	M	80	C	progressive necrosis of the right foot
DFI-2	F	63	C	necrosis of podfoot
DFI-3	M	69	P	phlegmon with gas-forming bacteria
DFI-4	F	83	C	right foot necrosis
DFI-5	F	83	C	right foot necrosis
DFI-6	M	72	Ci	right foot necrosis
DFI-7	M	55	C + L	phlegmon of the right foot
DFI-8	M	71	No	right foot necrosis
DFI-9	F	66	V + P	progressive metatarsal necrosis
DFI-10	M	61	Ci + P	finger amputation
DFI-11	M	56	No	phlegmon of the left leg
DFI-12	M	63	No	deep ulceration of the left heel with necrotic symptoms
DFI-13	M	81	C	dry necrosis of the toes of the left foot
DFI-14	F	64	C	purulent necrosis of the right stump
DFI-15	M	70	A	right foot necrosis
DFI-16	M	63	No	right foot phlegmon
DFI-17	F	85	Ci	extensive area of diffuse skin necrosis
DFI-18	M	73	A	dry toe necrosis of the right foot
DFI-19	M	52	C	right foot phlegmon
DFI-20	M	74	No	toe necrosis of the right foot
DFI-21	M	68	No	purulent necrosis of the left stump
DFI-22	M	63	Ci + C	right foot phlegmon
DFI-23	M	62	A	right foot phlegmon
DFI-24	M	62	No	toe necrosis of the left foot
DFI-25	M	62	C	deep phlegmon of the soft tissue of the sole of the left foot
DFI-26	M	62	No	toe and metatarsal bones necrosis of the left foot
DFI-27	M	54	No	toe necrosis of the right foot
DFI-28	M	66	C + A	toe necrosis of the right foot
DFI-29	M	42	No	left foot phlegmon
DFI-30	M	59	C	left foot phlegmon
DFI-31	M	45	A	right foot phlegmon

## Results

### Microbial Pattern of the DFI Samples Obtained via MALDI TOF MS

**Fig. 1:**
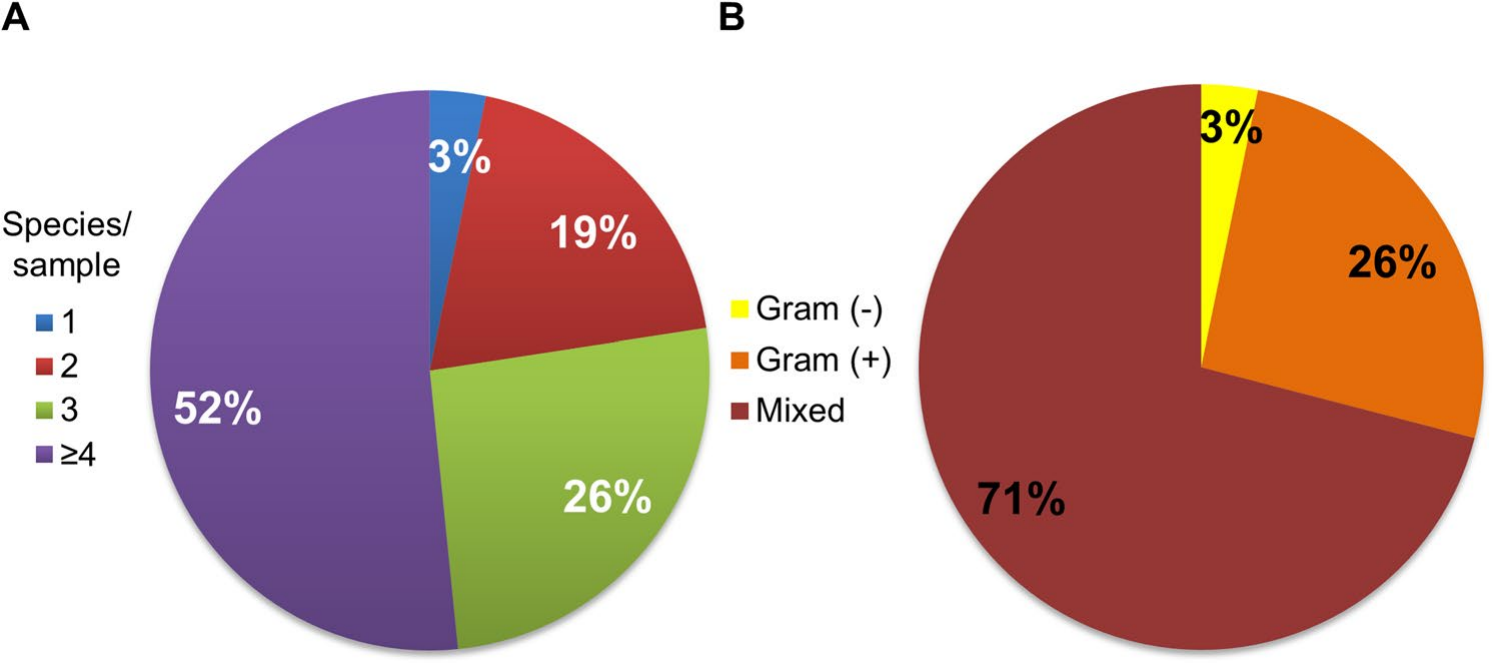
Pie charts presented frequency of the mono- and polymicrobial infections (**a**) as well as type of the bacteria (**b**) within samples collected from DFI patients

Applied MALDI protocol revealed that far most of the DFI patients suffered from polymicrobial infections—97% (Fig. 1a). Moreover, half of the infected wounds contained four or more different microbial species simultaneously – 52%. Over two-thirds of the wound samples were occupied by both Gram-positive and -negative species (71%), while another 26% by only Gram-positive (Fig. 1b). In two cases (3%) only Gram-negative species have been found.

**Fig. 2:**
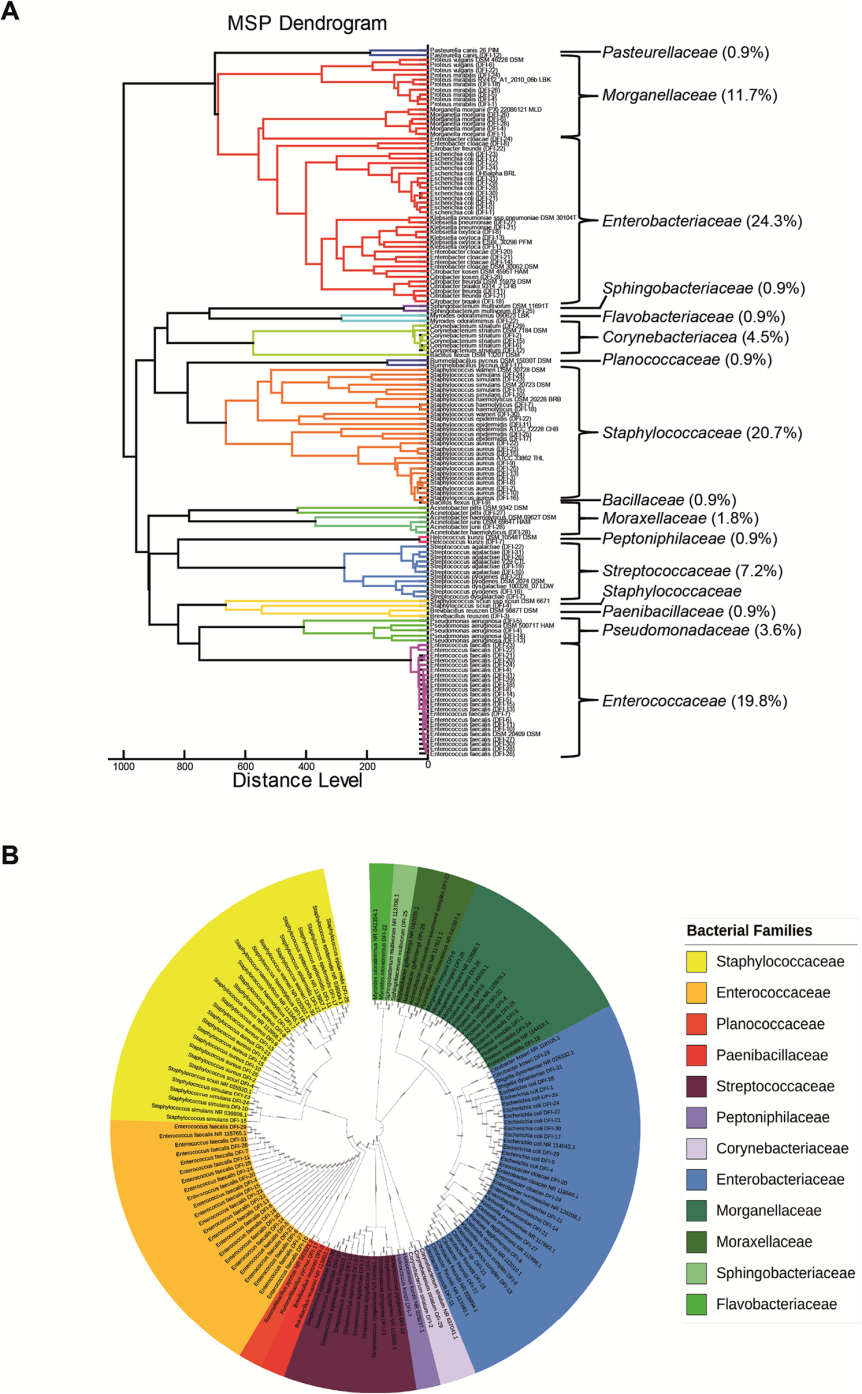
The results of the DFI microbiota deciphering using (**a**) MALDI TOF MS identification and (**b**) 16S rDNA sequencing technique

The result of the isolation step obtained 111 different bacterial isolates and two *Candida* species—*C. albicans* and *C. krusei* (patient DFI-22; Supplementary Table S1). Based on the MALDI identification, bacterial isolates represented 19 genera belonging to 16 different families—8 G( − ) and 7 G( +) (Fig. 2a). Among the most abundant groups that comprise almost two-thirds of all identified bacteria were *Enterobacteriaceae* (24.3%), *Staphylococcaceae* (20.7%), and *Enterococcaceae* (19.8%), represented mainly by *Enterococcus faecalis* (19.8%), *Escherichia coli* (10.8%), and *Staphylococcus aureus* (9.0%). Other frequently isolated species were *Proteus mirabilis* (5.4%), *Pseudomonas aeruginosa* (3.6%) as well as *Morganella morganii*, *Enterobacter cloacae*, *Streptococcus agalactiae*, and *Corynebacterium striatum* – each 4.5%.

### 16S rDNA Sequencing Results

We performed sequencing of the 16S rDNA region to evaluate the correctness of the MALDI identifications. In the results, 99 bacterial species have been identified—93% with species confidence (Supplementary Table S1). For 12 isolates, we do not get sequencing results due to strain loss during passaging (8 isolates) or the lack of a specific PCR product—the case of all *P.* *aeruginosa* strains. In the case of two isolates – *Klebsiella oxytoca* DFI-13 and *Citrobacter freundii* DFI-21—the analysis of the 16S rDNA region did not allow for a reliable determination of the species. In 7 cases, discrepancies between MALDI and 16S rDNA sequencing results have been noted—all of these concerned closely related species: *E. coli/S. dysenteriae*, *E. cloacae/hormaechei*, *C. freundii/braakii*, *E. cloacae/P. agglomerans*, *P. vulgaris/terrae*, and *Acinetobacter haemolyticus/gyllenbergii*. All in all, obtained MALDI identification was characterized by 98% genus and 93% species confidence, referring to the DNA sequencing results. Phylogenetic relationship of the identified bacterial isolates is presented on the Fig. 2b.

### Antibiotic Resistance Occurrence within Gram-Negative Isolates

**Fig. 3:**
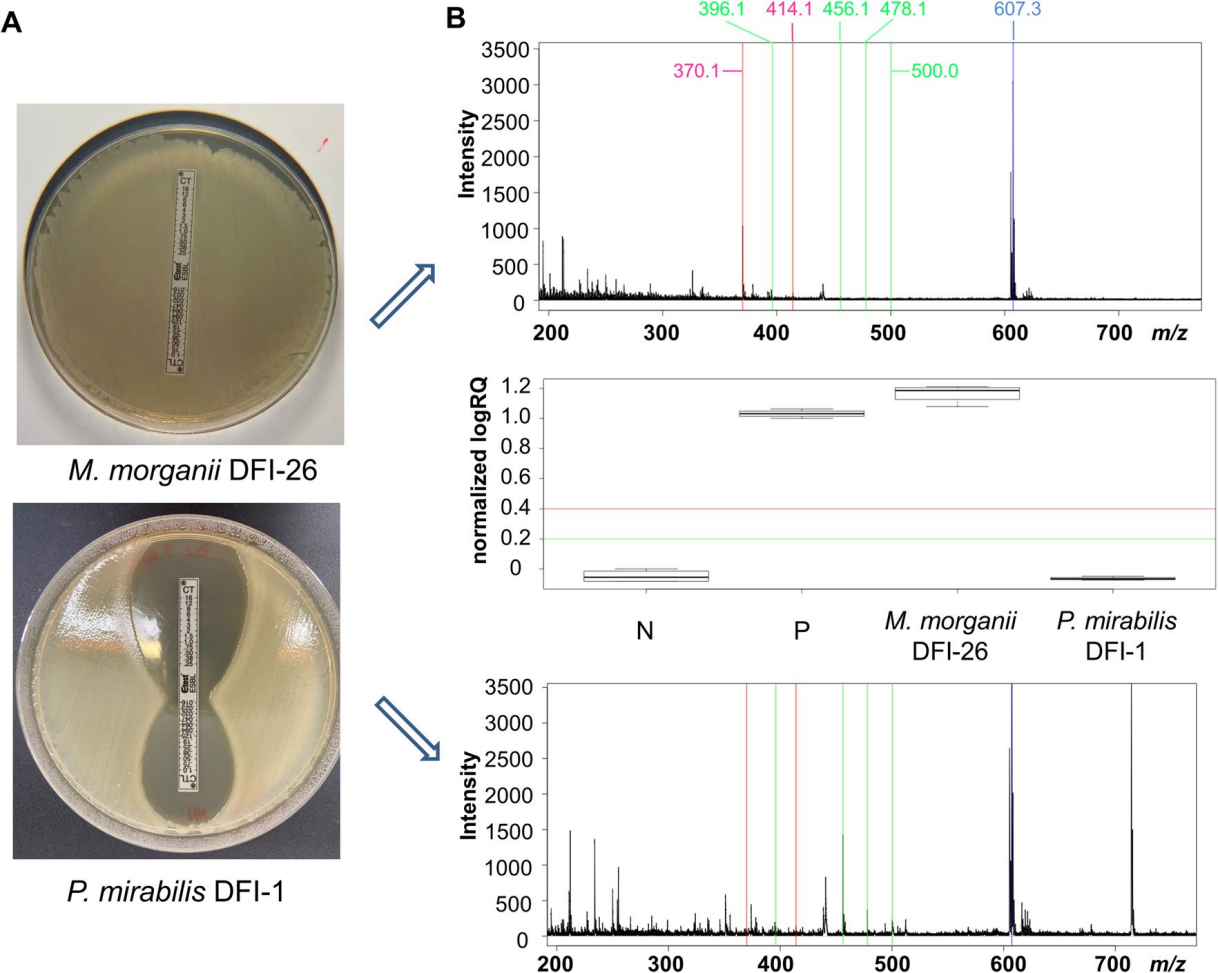
Exemplary results of antibiotic resistance detection performed using Etest strips (**a**) and MBT STAR BL method (**b**) obtained for cefotaxime. N- negative control, P—positive control, normalized logRQ values ≤ 0.2 threshold—negative results, ≥ 0.4 threshold—positive results, values between these thresholds—unclear results, green lines (396.1, 456.1, 478.1, 500.0)—signals for nonhydrolyzed antibiotic (native and adducts with HCCA); red lines (370.1 and 414.1)—signals for hydrolyzed antibiotic, blue line (607.3)—standard

Applying both MBT STAR BL assay and Etest strips enabled the detection of different types of antibiotic resistance among Gram-negative isolates examined. The examples of the Etest results and corresponding MALDI ones are presented in Fig. 3.

Regarding MALDI results, 16 out of 21 samples from DFI patients were occupied by Gram-negative bacteria that demonstrated resistance against at least one of the analyzed antibiotics (Table 3). The most frequent resistance against ampicillin was recorded – among 71% of patients and 52% of the isolates. Subsequently, over half of the patients (52%) showed the presence of ESBL strains covering common *Enterobacterales* species, such as *E. coli*, *C. freundii*, *K. oxytoca*, *M. morganii*, and *P. vulgaris*, as well as rarely founded in DFI samples—*Flavobacteriaceae* (*M. odoratimimus*) and *Sphingobacteriaceae* (*S. multivorum*) members. Considering ESBLs, for all of them, hydrolytic activity against cefotaxime was detected, while in the case of ceftriaxone and ceftazidime percentage of positive results among resistant strains dropped to 38.5 and 15.4%, respectively. Only for one strain – *C. koseri* DFI-28, the performed MBT STAR BL assay gave unclear results – values between 0.2 and 0.4. Referring to other antibiotics, 29% of strains (mainly *E. coli* and *P. mirabilis*) were resistant to piperacillin, while only 10% (*P. aeruginosa* DFI-5, *K. oxytoca* DFI-8, *M. odoratimimus* DFI-22, and *S. multivorum* DFI-25) showed enzymatic activity against carbapenems (imipenem or meropenem). The frequency of the occurrence of the specific drug resistance had been decreasing in the following order: ampicillin > ESBL > piperacillin > carbapenems. Considering results of the Etest strips, performed tests revealed higher percentage of ampicillin and piperacillin resistant strains compared to the MALDI ones – 67% and 50% Gram-negative strains, respectively. In the case of ESBL and carbapenemases-producing bacteria (CPB), the opposite observation had been noted—the share of ESBL strains was lower by 10% and for CPB by 8%. The number of unclear (undefined) results also significantly increases when Etest strips are used, which mostly refers to ESBL detection—cefotaxime 6/42 (14%) and other cephalosporins: ceftazidime 4/42 (10%), cefepime 3/42 (7%), and ceftriaxone 1/42 (2%). Unlike to MALDI results, the highest number of ESBL activity was detected using ceftriaxone which covered two-thirds of all positive strains based on the Etest strips test. In the case of other cephalosporins, the percentage of positive results ranged from 33 to 42% mostly due to the high rate of undefined results.

**Table 3:**
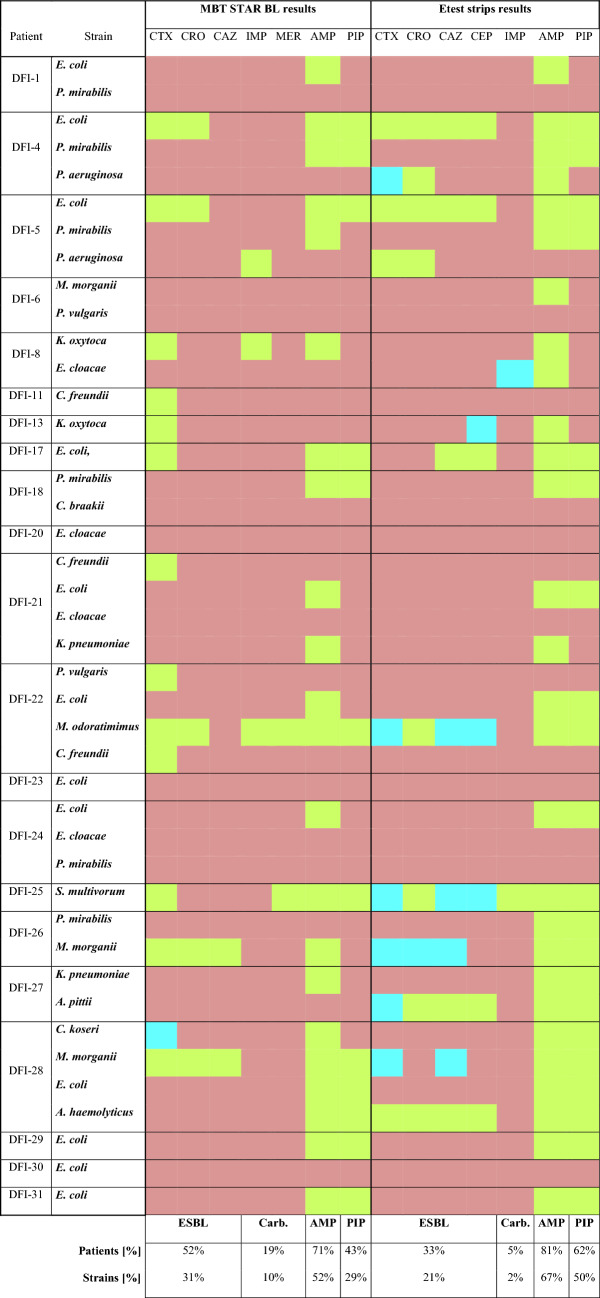
Occurrence of the different types of antibiotic resistance among gram-negative isolates that were detected using MALDI and Etest strips approaches

Green—positive results, purple—negative results, blue—unclear results, CTX—cefotaxime, CRO—ceftriaxone, CAZ—ceftazidime, CEP—cefepime, IMI—imipenem, MER—meropenem, AMP—ampicillin, PIP—piperacillin, ESBL—extended spectrum β-lactamases, Carb.—carbapenemases

Regarding specific antibiotics, the far most significant number of discrepancies between the two methods used were noted for cefotaxime – 16, which resulted from a higher rate of negative and undefined outcomes of Etests. Contrary to this, ampicillin hydrolysis detection was characterized by the highest complies level—~ 76%. Regarding bacterial species, the most different results were observed in the case of *P. aeruginosa* strains and *Citrobacter* and *Acinetobacter* genera members. Numerous discrepancies were also noted for *M. odoratimimus* and *S. multivorum, *which could be associated with their high enzymatic activity leading to undefined results in the Etests.

Screening for antibiotic-resistance genes revealed that only ten strains possessed one or more of the beta-lactamase-encoding genes tested (Table 4). They all refer to ESBLs representing Ambler class A beta-lactamases—TEM, SHV, or CTX-M. Most strains carrying resistance genes were *E. coli* strains (7) that most frequently had *TEM* (5 isolates). Regarding other genes, *SHV* occurred three times – in *K. oxytoca* DFI-8, *E. coli* DFI-17, and *K. pneumoniae* DFI-21, while *CTX-M-9* 2 times – *E. coli* DFI-4 and -5. Interestingly, only in 4 cases were ESBL genes detected for strains that demonstrated resistance according to MALDI or Etest assays – *E. coli* DFI-4 possesses *CTX-M-9*, *E. coli* DFI-5 with *TEM* + *CTX-M-9* as well as *K. oxytoca* DFI-8 and *E. coli* DFI-17 – both with *SHV* like genes. The rest strains with detected resistant genes demonstrated only activity against ampicillin or piperacillin and mostly had *TEM* – 5 out of 6 isolates. Despite the detection of carbapenemase activity in 4 strains, PCR analyses did not reveal the presence of any of the analyzed genes encoding carbapenemases, that is, *VIM*, *IMP*, *NDM*, *GIM*, *KPC*, *GES*, or *OXA-48*.

**Table 4:**
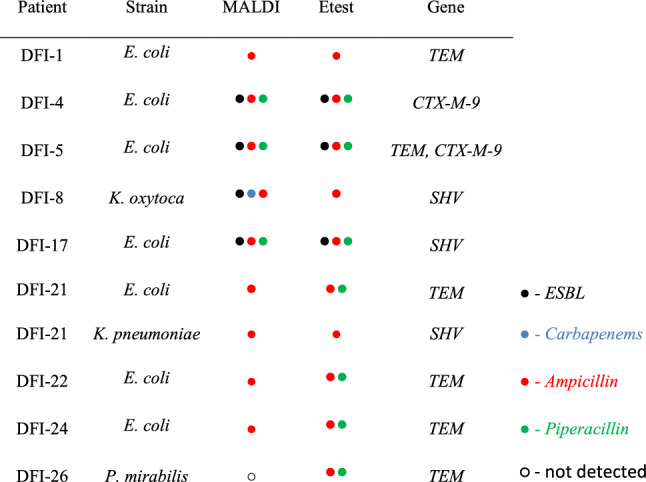
List of strains in which the tested resistance genes were detected using established multiplex PCR protocols with marked resistance according to MBT STAR BL and Etest

### Impact of the Antibiotic Therapy on the Microbiological Outcome of the DFI Patients

**Fig. 4:**
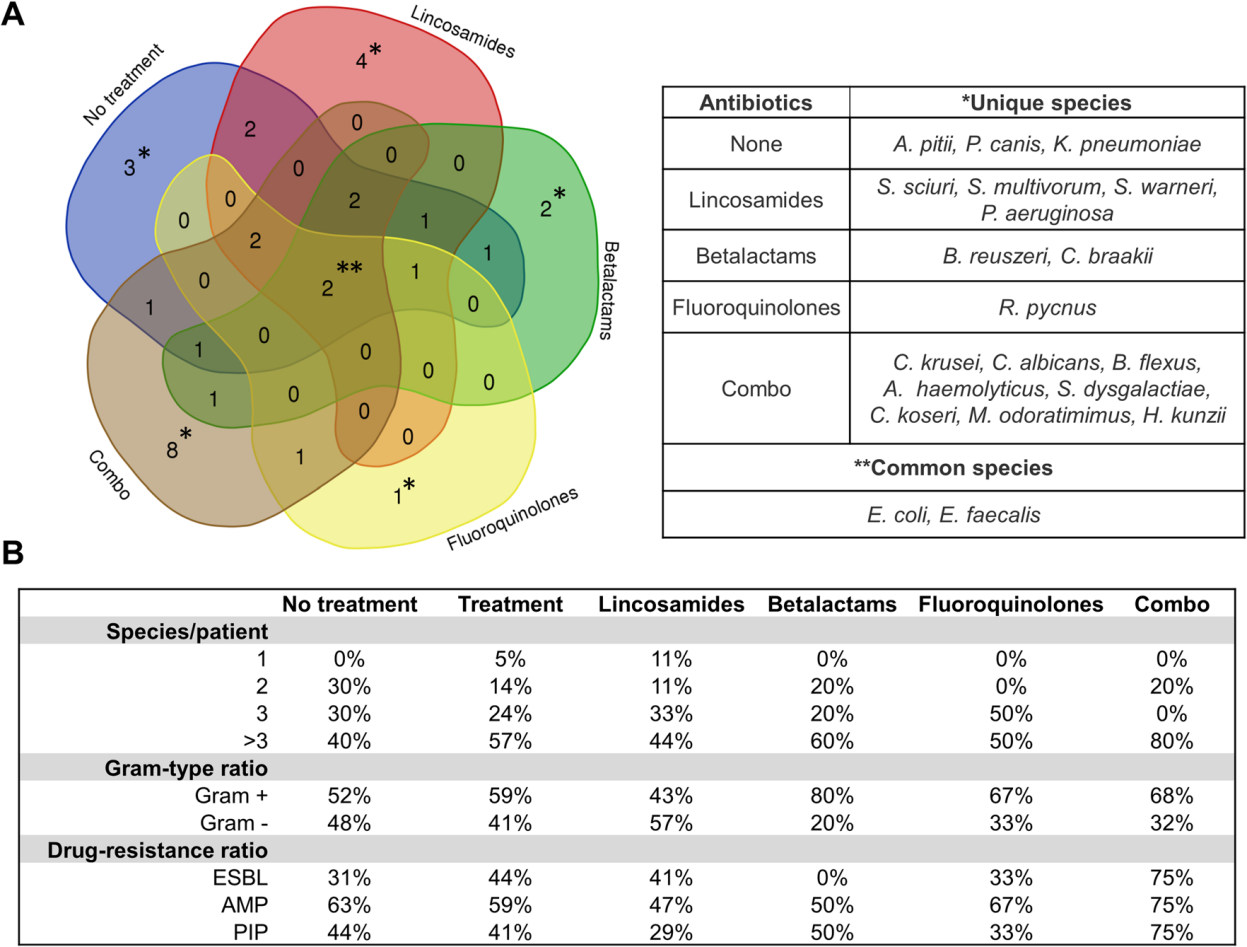
Effect of the antibiotic treatment on the microbial profiles of the swab samples of the DFI patients. **a**—Venn diagram showing unique/common bacterial species depending on the antibiotic type used. **b**—influence of the treatment on the number of species per patient, Gram-type ratio as well as occurrence of the antibiotic resistance (ESBL—extended-spectrum beta-lactamase, AMP—ampicillin resistance, PIP—piperacillin resistance). Resistance against carbapenems was not presented due to poor representation among investigated samples

According to medical history, 10 DFI patients were not subjected to any antibiotic treatment; nine were receiving lincosamides (clindamycin), five beta-lactams, two fluoroquinolones, and five were receiving multi-antibiotic treatment (combo). Comparison of the species composition of swab samples according to the antimicrobial treatment used revealed differences in the type of microbial species detected that were considered unique to each patient group (Fig. 4a). Venn diagram analysis showed that when a particular type of antibiotic was used, the species composition of the DFI samples changed. The number of species highlighted varied depending on the type of antibiotic used. The highest number of unique microbial species (8) was found in combined antibiotic treatment, including two *Candida* species, and in patients treated with lincosamides—5. In contrast, samples from patients treated with beta-lactams and fluoroquinolones had fewer specific species, 2 and 1, respectively, but this observation may be due to the lower representation of these groups. Two bacteria, *E. coli*, and *E. faecalis*, appeared to be species common to all groups of DFI patients.

The analysis showed that the antibiotic treatment also affected the number of microbial species presents simultaneously in the sample; samples from treated patients had a higher percentage of samples with four or more microbial species—57% versus 44% (Fig. 4b). Such phenomenon was particularly evident in samples from patients on combination antibiotic therapy, where the percentage of samples containing > 3 species in the sample reached 80%. Considering the prevalence of a particular type of bacteria, antibiotic therapy generally increased Gram-positive bacteria, except for lincosamides, where the opposite trend was observed. A tremendous increase in Gram-positive bacteria was observed with beta-lactams—80% compared to 52% in the untreated group. Samples from treated patients were generally characterized by a higher ESBL share – 44% compared to 31% in the non-treated patients, but a slightly lower percentage of the ampicillin and piperacillin-resistant strains occurrence. The highest percentage of drug-resistant Gram-negative bacteria was observed in DFI patients receiving combination antibiotic therapy, with 75% resistant to ESBLs, ampicillin, and piperacillin.

## Discussion

Optimal treatment of the infection depends on accurately identifying the microorganisms present and applying appropriate antimicrobial treatment. Failure to adequately treat infection in diabetic foot ulcers leads to progressive tissue damage, impaired wound healing, and serious complications [28]. Clinical practice of DFI diagnosis has relied chiefly on cultivation-dependent methods, which show bias towards microorganisms that thrive under isolation procedures and can grow well on laboratory culture media. Therefore, they often overlook slow-growing, fastidious, anaerobic, and unknown pathogens, which delays the appropriate treatment [29]. In the literature, we can find numerous examples of studies using the traditional culture method, where 46–85% of DFI cases were monobacterial [30, 31] with only a minority being polymicrobial infections [32].

On the other hand, there are also papers in which authors have indicated that DFIs are more often the result of polymicrobial infection with complex bacterial communities (microbiome) that impede wound healing [33]. More recent advances in molecular biology technologies have helped to overcome obstacles accompanying traditional methods providing new insights into the bacterial diversity of DFI and have confirmed that chronic wounds, including diabetic foot ulcers, have a polymicrobial nature instead of being colonized by a single species [34, 35]. Price et al. [36] found that culture-based method revealed only nine bacterial families compared to 44 denoted using 16S rRNA sequencing which may be the reason for the high prevalence of monomicrobial infections detected by traditional culture. Nevertheless, it should be noticed that molecular approaches are limited by amplification biases, namely, by the primer choice affecting the amplification efficiency of different microbial phyla, as well as by the quality of extracted DNA which depends on the microbial taxa [37]. The optimal culture conditions selected in our previous study [8] enabled revealing a large diversity of bacterial families involved in the development of DFI including rarely detected in DFI *Pasteurellaceae*, *Sphingobacteriaceae*, *Flavobacteriaceae*, *Planococaceae*, and *Peptoniphilaceae*.

Contrary to popular belief that cultures have a high false-negative rate and lack full representation of the total microbial population in wounds, especially in terms of the pathogenic burden [38, 39], culture-based methods can still play an essential role in patient management providing that modern culturomics approach with rapid microbial identification via MALDI technique is applied. As shown in our study, the simultaneous use of culture media sets of different types (universal, selective/differentiating) allows the isolation of fast-growing bacterial species as well as fastidious ones represented both Gram-positive and –negative type of bacteria in short time-to-results. Furthermore, the MALDI technique application assured high identification confidence by comparing species level with 16S rDNA sequencing – 93%. To date, only two papers have been published in this field regarding DFI research—our previous work concerning selection culture conditions [8]. and work Jneid et al. [40]. In the second case, authors found a high prevalence of polymicrobial infections (88.3%) and high biodiversity (53 known and 19 unknown bacterial species). In addition, the culture conditions used allowed the isolation of species commonly found in DFI (mostly *S. aureus*, *Enterococcus faecalis*, *Enterobacter cloacae*) as well as the rarest species, such as anaerobic *Finegoldia magna*. Both studies mentioned above have proven that culturomics does work as a solution to address the limitations of conventional culturing, that is, increase the throughput of identifications and species coverage as well as play a complementary role concerning molecular methods in the exploration of complex microbiota in DFIs.

Revealed high frequency of polymicrobial infections is of utmost importance for patient management since microbial interactions may synergize the pathogenic potential of one or other microorganism, hampering their eradication and further controlling chronic wounds [38]. Liu et al. [4] hypothesized that individual bacterial species may not be able to maintain a pathogenic biofilm independently. However, pathogenic biofilm formation may occur in a symbiotic polymicrobial community in the DFU. Therefore, although most of our studies, *Staphylococcus* spp., and *Corynebacterium* spp., are considered part of healthy skin's normal microbiota, they may contribute to a pathogenic community of DFI. Several studies have highlighted the importance of CoNS and *Corynebacterium* spp. as potential pathogens of DFI and stress their importance concerning chronic wounds, especially in the case of patients with impaired immune responses such as diabetes Our studies revealed a significant share of bacteria belonging to *Enterobacteriaceae* – 24.3% of all identified bacterial families and correlated with other culture-based studies that reported a high incidence of *Enterobacteriaceae* members in moderate to severe diabetic foot ulcers [38, 41]. The predominance of the *Enterobacteriaceae* family has recently been reported as the largest group of aerobic Gram-negative rods in DFIs [42]. A shift towards the presence of enteric types of bacteria in the recurrent wound may be a result of self-colonization from another body site, e.g., gastrointestinal tracts, which produced a corresponding decline in wound healing since many of such Gram-negative isolates may also be multidrug-resistant which makes them very difficult to eradicate with antibiotic therapy. Indeed, drug resistance analysis among isolated Gram-negative bacterial strains showed a relatively high prevalence of ESBL (52% of isolates and 31% of patients) and carbapenemase-producing bacteria—19% of all Gram-negative isolates. Because the increasing severity of prevention and treatment of diabetic foot ulcer infections is associated with high rates of detection of multidrug-resistant bacteria, it is crucial to focus on assessing risk factors for infection with multidrug-resistant bacteria to find more effective treatments [42, 43]. Yan et al. [43] during the analysis of risk factors for multidrug-resistant organisms in diabetic foot infection among 180 patients from the Hospital of Jiangnan University (Wuxi Area), noted that 104 of all 182 isolated strains were multidrug-resistant bacteria (66 strains of Gram-negative bacteria and 38 strains of Gram-positive bacteria). In addition, the authors noted that antimicrobial use in the past 3 months was associated with multidrug-resistant bacterial infections in patients with diabetic foot ulcers (*P* < 0.05).

The microbial load, diversity, and presence of pathogenic organisms in the DFU are known to change in response to antibiotic treatment [44]. It has been noted that the wound microbiota of patients treated with antibiotics is significantly different from that of untreated patients. However, no clear distinction has been made between problematic bioburden and benign colonization, which would be clinically relevant to antibiotic treatment decisions [44, 45]. Our results showed that the use of antibiotic therapy by patients induces changes in the microbial composition and frequency of species in the wound microbiota, including the gram-type ratio and the frequency of drug resistance. The proportion of Gram-negative bacteria decreased while the antibiotic resistance rate increased. This observation of combination antibiotic therapy was remarkably accurate, indicating the highest number of unique microbial species and the highest ratio of drug-resistant strains. The resistance rate of *E. coli* was the highest among Gram-negative bacteria, consistent with previous reports. This founding may also be related to the fact that *E. coli* was also reported as the most common Gram-negative bacterium, as in many other reports, e.g. Tascini et al. [46].

Infection with MDR bacteria in DFU reduces the clinical effect of antibiotic therapy. Our study indicates that empirical antibiotic therapy for DFI should pay particular attention to the risk assessment of Gram-negative bacteria infection, where the susceptibility pattern of Gram-negative bacteria should be regularly monitored in DFI. Nevertheless, many clinics rely on traditional culture and conventional biochemical tests, like strip test, that underestimates wound flora and may lead to inappropriate antibiotics prescribed in up to 45% of cases [47]. The excessive or inappropriate use of antibiotics not only results in ineffective treatment but also aggravates the worldwide crisis of antibiotic resistance [38]. This problem could be solved by using the latest, more adequate, and rapid drug resistance assays, such as those based on the MALDI TOF MS technique. Our study showed that using the MBT STAR BL assay increased the percentage of ESBL- and carbapenemase-producing bacteria detected compared to Etest strips. Additionally, the use of MBT STAR BL is accompanied by a significantly lower rate of unclear results. More and more researchers, including Noster et al. [48], emphasize that the MALDI technique is increasingly embraced for detecting antimicrobial resistance and will likely become an essential part of the routine laboratory soon. Although the disadvantage of this approach is that it only detects resistance conferred by hydrolysis of the target antibiotic, it has high sensitivity and specificity (98–100% and 97–100%, respectively), as well as a relatively short turnaround time—usually 30 min for typical Enterobacterales, and even shorter if an appropriate protocol modification is used, as demonstrated by Złoch et al. [21]. As our studies showed, the application of the MALDI approach could be more feasible for routine drug resistance detection among DFI isolates than molecular technique, such as multiplex PCR reactions, since the latter required expanded knowledge about the taxonomical affiliation of the isolates for suitable primers set designing.

## Conclusions

Reliable deciphering of the composition of the wound microbiome in patients with DFI is crucial for subsequent effective therapy. Given the large number of microbial species that may be involved in the development of infection, especially in moderate to severe chronic wounds, practical diagnostic tools should be characterized by accurate identification, short time-to-results as well as the ability to rapidly detect drug resistance in the face of a growing global problem such as MDR bacteria. Such criteria are met by fast MALDI identification combined with multiple culture conditions and rapid detection of antibiotic resistance via MBT STAR BL assay. As demonstrated in our study, this method provides identification information at a level comparable to that obtained from DNA sequencing, allows the isolation of both common bacterial species and those considered rare, including fastidious ones, and is effective in antibiotic detection, especially for that of particular concern like ESBLs and carbapenemases. Application of this technique may help to understand the role of the complex microbiota in the development of DFI in the context of the antibiotic therapy used by patients and its impact on the development of drug resistance. Moreover, our results indicate that culture-based methods can still be essential to routine clinical diagnosis, providing the clinician with relevant information in a reasonable time.

## Supplementary Information

Below is the link to the electronic supplementary material.

Supplementary file1 (DOCX 39 kb)

## Data Availability

All sequence data that support the findings of this study have been deposited in GenBank and the public URL for the sequences collection is https://www.ncbi.nlm.nih.gov/sites/myncbi/1F1i8raBplj5d/collections/61834280/public/

## Code Availability

Software for DNA sequences analysis: MEGA software version 7.0.21; BioEdit Sequence Alignment Editor version 7.2.5 (12/11/2013); Interactive Tree of Life (iTOL) version 6.5.4 (01/09/2021). Software for MALDI TOF MS analysis: flexControl version 3.4 (Build 135); flexAnalysis version 3.4 (Build 76); MALDI Biotyper Compass Explorer 4.1 (Build 80), Bruker MBT STAR-BL Prototype (RUO) https://mbtprot.bdal.de/MSBL/ (private access).
