# Supplementary material for: Multi-Instrumental Analysis Toward Exploring the Diabetic Foot Infection Microbiota
Source: Curr Microbiol. 2023 Jul 5;80(8):271. doi: 10.1007/s00284-023-03384-z (PMC10322772; doi:10.1007/s00284-023-03384-z)
Supplement: Supplementary file 1 — Supplementary file1 (DOCX 39 kb) [file 284_2023_3384_MOESM1_ESM.docx]

Supplementary

**Supplementary Tables captions:**

**Supplemenatary Table S1.** List of the identified isolates with the Score values (MALDI TOF MS) and percentage of identity with the NCBI records (16S rDNA sequencing) with obtain accession numbers.

**Supplemenatary Table S2.** Temperature program used for specific primers sets in order to detect investigated antibiotic-resistance genes.

**Supplemenatary Table S1.**

| **Patient** | **MALDI** | | | | **16S rDNA sequencing** | **Identity**  **[%]** | **Accession no.** |
| --- | --- | --- | --- | --- | --- | --- | --- |
|  | **RAW** | | **MSP** | | **Related species from NCBI*** |  |  |
| DFI-1 | *Escherichia coli* DH5alpha BRL | 2,21 | *Escherichia coli* DH5alpha BRL | 2,27 | *Escherichia coli* NBRC 102203 | 99,34 | MZ914687 |
|  | *Klebsiella oxytoca* VA20876_2_09 ERL | 2,08 | *Klebsiella oxytoca* VA20876_2_09 ERL | 2,04 | *-* |  |  |
|  | *Morganella morganii* 9544_1 CHB | 2,01 | *Morganella morganii* 9544_1 CHB | 2,34 | *-* |  |  |
|  | *Proteus mirabilis* DSM 788 DSM | 2,09 | *Proteus mirabilis* DSM 788 DSM | 2,41 | *Proteus mirabilis* ATCC 29906 | 99,86 | MZ914688 |
| DFI-2 | *Corynebacterium striatum* MB_8820_05 THL | 2,37 | *Corynebacterium striatum* 10_G1799 ISB | 2,44 | *Corynebacterium striatum* ATCC 6940 | 99,54 | MZ914698 |
|  | *Staphylococcus aureus* ATCC 33591 THL | 2,39 | *Staphylococcus aureus* ATCC 33591 THL | 2,30 | *Staphylococcus aureus* NBRC 100910 | 99,93 | MZ915637 |
| DFI-3 | *Brevibacillus reuszeri* DSM 9887T DSM | 1,92 | *Brevibacillus reuszeri* DSM 9887T DSM | 1,84 | *Brevibacillus reuszeri* NBRC 15719 | 100 | MZ918943 |
|  | *Staphylococcus aureus ssp aureus* DSM 20232 DSM | 2,44 | *Staphylococcus aureus* ATCC 29213 THL | 2,47 | *-* |  |  |
| DFI-4 | *Enterococcus faecalis* ATCC 7080 THL | 2,12 | *Enterococcus faecalis* ATCC 7080 THL | 2,12 | *Enterococcus faecalis* ATCC 19433 | 100 | MZ918944 |
|  | *Escherichia coli* ATCC 35218 CHB | 2,20 | *Escherichia coli* DH5alpha BRL | 2,14 | *Escherichia coli* NBRC 102203 | 99,72 | MZ918945 |
|  | *Morganella morganii ssp morganii* DSM 30164T HAM | 2,39 | *Morganella morganii ssp morganii* DSM 30164T HAM | 2,42 | *-* |  |  |
|  | *Proteus mirabilis* 13210_1 CHB | 2,03 | *Proteus mirabilis* DSM 30115 DSM | 2,19 | *Proteus mirabilis* JCM 1669 / ATCC 29906 | 99,79 | MZ918947 |
|  | *Staphylococcus sciuri ssp sciuri* DSM 20345T DSM | 2,11 | *Staphylococcus sciuri ssp sciuri* DSM 20345T DSM | 2,18 | *Staphylococcus sciuri* DSM 20345 | 99,86 | MZ918949 |
|  | *Pseudomonas aeruginosa* 8147_2 CHB | 2,31 | *Pseudomonas aeruginosa* DSM 50071T HAM | 2,31 | ? |  |  |
| DFI-5 | *Enterococcus faecalis* ATCC 7080 THL | 2,27 | *Enterococcus faecalis* ATCC 7080 THL | 2,21 | *Enterococcus faecalis* ATCC 19433 | 100 | MZ918965 |
|  | *Escherichia coli* MB11464_1 CHB | 2,11 | *Escherichia coli* MB11464_1 CHB | 2,25 | *Escherichia coli* NBRC 102203 | 99,72 | MZ918988 |
|  | *Proteus mirabilis 13210_1 CHB* | 2,22 | *Proteus mirabilis* 13210_1 CHB | 2,34 | *Proteus mirabilis* ATCC 29906 | 99,86 | MZ919118 |
|  | *Pseudomonas aeruginosa* 8147_2 CHB | 2,19 | *Pseudomonas aeruginosa* 8147_2 CHB | 2,13 | ? |  |  |
| DFI-6 | *Corynebacterium striatum* MB_8820_05 THL | 2,38 | *Corynebacterium striatum* MB_8820_05 THL | 2,49 | - |  |  |
|  | *Enterococcus faecalis* ATCC 7080 THL | 2,37 | *Enterococcus faecalis* DSM 6134 DSM | 2,40 | *Enterococcus faecalis* ATCC 19433 | 100 | MZ919144 |
|  | *Morganella morganii ssp sibonii* Mb19277_2 CHB | 2,54 | \| *Morganella morganii ssp sibonii*  Mb19277_2 CHB \| \| --- \| | 2,49 | *Morganella morganii subsp. sibonii* DSM 14850 | 99,78 | MZ919317 |
|  | *Proteus vulgaris* DSM 30119 DSM | 1,98 | *Proteus vulgaris* DSM 46228 DSM | 2,20 | *Proteus terrae* N5/687 | 100 | MZ919328 |
| DFI-7 | *Enterococcus faecalis* ATCC 7080 THL | 2,36 | *Enterococcus faecalis* ATCC 29212 CHB | 2,28 | *Enterococcus faecalis* ATCC 19433 | 100 | MZ919328 |
|  | *Streptococcus dysgalactiae ssp dysgalactiae* DSM 20662T DSM | 2,45 | *Streptococcus dysgalactiae* VA20336_10 ERL | 2,36 | *Streptococcus dysgalactiae subsp. equisimilis* CIP 105120 | 99,93 | MZ919346 |
|  | *Staphylococcus haemolyticus* 10024 CHB | 2,57 | *Staphylococcus haemolyticus* 10024 CHB | 2,25 | *Staphylococcus haemolyticus* JCM 2416 | 99,93 | MZ919344 |
|  | *Helcococcus kunzii* VA324_10 ERL | 2,45 | *Helcococcus kunzii* VA324_10 ERL | 2,42 | *Helcococcus kunzii* 22 | 98,64 | MZ919347 |
| DFI-8 | *Enterococcus faecalis* ATCC 7080 THL | 2,28 | *Enterococcus faecalis* ATCC 7080 THL | 2,23 | *Enterococcus faecalis* ATCC 19433 | 100 | MZ919357 |
|  | *Staphylococcus aureus* ATCC 29213 THL | 2,38 | *Staphylococcus aureus* ATCC 29213 THL | 2,28 | *Staphylococcus aureus* NBRC 100910 | 99,00 | MZ919968 |
|  | *Klebsiella oxytoca* VA20879_09 ERL | 2,16 | *Klebsiella oxytoca* VA31877_09 ERL | 2,13 | *Klebsiella oxytoca* JCM 1665 | 99,43 | MZ919988 |
|  | *Enterobacter cloacae* DSM 3264 DSM | 2,20 | *Enterobacter ludwigii* DSM 16688T DSM | 1,66 | *Pantoea agglomerans* JCM1236 | 99,71 | MZ919989 |
| DFI-9 | *Staphylococcus aureus ssp aureus* DSM 20232 DSM | 2,37 | *Staphylococcus aureus* ATCC 33591 THL | 1,80 | *Staphylococcus aureus* NBRC 100910 / ATCC 12600 | 100 | MZ920051 |
|  | *Bacillus flexus* 100331_30 USP | 2,09 | *Bacillus flexus* 100331_30 USP | 2,15 | *-* |  |  |
| DFI-10 | *Enterococcus faecalis* DSM 20371 DSM | 2,29 | *Enterococcus faecalis* ATCC 7080 THL | 2,20 | *Enterococcus faecalis* ATCC 19433 | 100 | MZ921477 |
|  | *Streptococcus agalactiae* DSM 6784 DSM | 2,33 | *Streptococcus agalactiae* DSM 6784 DSM | 2,33 | *Streptococcus agalactiae* ATCC 13813 | 100 | MZ921512 |
|  | *Staphylococcus simulans* DSM 20723 DSM | 1,81 | *Staphylococcus simulans* DSM 20723 DSM | 1,71 | *Staphylococcus simulans* MK 148 | 99,93 | MZ921511 |
|  | *Staphylococcus aureus* ATCC 33862 THL | 2,27 | *Staphylococcus aureus ssp aureus* DSM 4910 DSM | 2,33 | *Staphylococcus aureus* NBRC 100910 | 100 | MZ921946 |
| DFI-11 | *Enterococcus faecalis* ATCC 7080 THL | 2,22 | *Enterococcus faecalis* DSM 20409 DSM | 2,21 | *Enterococcus faecalis* ATCC 19433 | 100 | MZ922053 |
|  | *Staphylococcus epidermidis* ATCC 14990T THL | 1,66 | *Staphylococcus epidermidis* ATCC 14990T THL | 1,73 | *Staphylococcus epidermidis Fussel* | 100 | MZ922259 |
|  | *Citrobacter freundii* 22054_1 CHB | 2,39 | *Citrobacter freundii* 22054_1 CHB | 2,42 | *Citrobacter freundii* LMG 3246 | 99,78 | MZ930404 |
| DFI-12 | *Corynebacterium striatum* MB_8820_05 THL | 2,48 | *Corynebacterium striatum* MB_8820_05 THL | 2,49 | ? |  |  |
|  | *Pasteurella canis* SO_02063_09 ERL | 1,97 | *Pasteurella canis* 26 PIM | 2,06 | *-* |  |  |
| DFI-13 | *Enterococcus faecalis* ATCC 29212 CHB | 2,38 | *Enterococcus faecalis* DSM 20371 DSM | 2,44 | *Enterococcus faecalis* ATCC 19433 | 100 | MZ930405 |
|  | *Staphylococcus aureus* ATCC 33591 THL | 2,28 | *Staphylococcus aureus* ATCC 33591 THL | 2,41 | *Staphylococcus aureus* NBRC 100910 | 100 | MZ930410 |
|  | *Pseudomonas aeruginosa* ATCC 27853 THL | 2,16 | *Pseudomonas aeruginosa* ATCC 27853 THL | 2,22 | *?* |  |  |
|  | *Klebsiella oxytoca* VA31877_09 ERL | 2,17 | *Klebsiella oxytoca* 35130 PFM | 2,29 | *Klebsiella oxytoca* JCM 1665/ *Klebsiella grimontii* SB73 | 99,28/ 99,21 | MZ930463 |
| DFI-14 | *Enterococcus faecalis* ATCC 7080 THL | 2,13 | *Enterococcus faecalis* DSM 2570 DSM | 2,29 | *Enterococcus faecalis* ATCC 19433 | 100 | MZ930473 |
|  | *Pseudomonas aeruginosa* ATCC 27853 THL | 2,09 | *Pseudomonas aeruginosa* DSM 50071T HAM | 2,10 | *?* |  |  |
|  | *Enterobacter cloacae* DSM 46348 DSM | 2,50 | *Enterobacter cloacae* DSM 46348 DSM | 2,39 | *Enterobacter hormaechei subsp. xiangfangensis* | 99,64 | MZ930474 |
| DFI-15 | *Enterococcus faecalis* ATCC 7080 THL | 2,32 | *Enterococcus faecalis* DSM 20409 DSM | 2,38 | *Enterococcus faecalis* ATCC 19433 | 100 | MZ930475 |
|  | *Staphylococcus simulans DSM 20723 DSM* | 1,91 | *Staphylococcus simulans* DSM 20723 DSM | 2,09 | *Staphylococcus simulans* MK 148 | 99,86 | MZ930477 |
|  | *Corynebacterium striatum* MB_8820_05 THL | 2,39 | *Corynebacterium striatum* MB_8820_05 THL | 2,44 | *-* |  |  |
|  | *Staphylococcus aureus ssp aureus* DSM 11822 DSM | 2,00 | *Staphylococcus aureus* ATCC 29213 THL | 1,95 | *Staphylococcus aureus* NBRC 100910 | 99,93 | MZ930479 |
| DFI-16 | *Streptococcus pyogenes* ATCC 19615 THL | 2,42 | *Streptococcus pyogenes* DSM 2072 DSM | 2,47 | *Streptococcus pyogenes* JCM 5674 | 100 | MZ930480 |
|  | *Staphylococcus aureus* ATCC 29213 THL | 2,36 | *Staphylococcus aureus* ATCC 33591 THL | 2,34 | *Staphylococcus aureus* NBRC 100910 | 100 | MZ931308 |
| DFI-17 | *Escherichia coli* DH5alpha BRL | 1,99 | *Escherichia coli* DH5alpha BRL | 2,20 | *Escherichia coli* NBRC 102203 | 99,50 | MZ931297 |
|  | *Rummeliibacillus pycnus* DSM 15030T DSM | 2,03 | *Rummeliibacillus pycnus* DSM 15030T DSM | 2,17 | *Rummeliibacillus pycnus* NBRC 101231 | 99,86 | MZ931298 |
|  | *Staphylococcus epidermidis* 10547 CHB | 2,18 | *Staphylococcus epidermidis* 10547 CHB | 2,38 | *Staphylococcus epidermidis Fussel* | 100 | MZ931299 |
| DFI-18 | *Enterococcus faecalis* ATCC 7080 THL | 2,35 | *Enterococcus faecalis* DSM 6134 DSM | 2,49 | *Enterococcus faecalis* ATCC 19433 | 99,93 | MZ931305 |
|  | *Proteus mirabilis* RV412_A1_2010_06b LBK | 2,46 | *Proteus mirabilis* RV412_A1_2010_06b LBK | 2,53 | *Proteus mirabilis* ATCC 29906 | 99,93 | MZ931326 |
|  | *Citrobacter braakii* 9314_2 CHB | 2,16 | *Citrobacter braakii* 20663_2 CHB | 2,31 | *Citrobacter freundii* LGM 3246 | 99,50 | MZ934698 |
|  | *Staphylococcus haemolyticus* 10024 CHB | 2,39 | *Staphylococcus haemolyticus* 10024 CHB | 2,28 | *Staphylococcus haemolyticus* JCM 2416 | 99,86 | MZ934700 |
| DFI-19 | *Streptococcus agalactiae* V29 CTL | 2,50 | *Streptococcus agalactiae* 03_102 CTL | 2,56 | *Streptococcus agalactiae* ATCC 13813 | 100 | MZ934701 |
| DFI-20 | *Enterobacter cloacae* MB_8779_05 THL | 2,11 | *Enterobacter cloacae* MB_8779_05 THL | 2,27 | *Enterobacter cloacae* ATCC 13047 | 99,56 | MZ934702 |
|  | *Enterococcus faecalis* 20247_4 CHB | 2,26 | Enterococcus faecalis DSM 6134 DSM | 2,46 | *Enterococcus faecalis* ATCC 19433 | 100 | MZ934703 |
| DFI-21 | *Enterococcus faecalis* ATCC 7080 THL | 2,08 | *Enterococcus faecalis* DSM 2570 DSM | 2,45 | *Enterococcus faecalis* ATCC 19433 | 100 | MZ934744 |
|  | *Citrobacter freundii* 22054_1 CHB | 2,27 | *Citrobacter freundii* 22054_1 CHB | 2,49 | *Citrobacter sp*  *(Citrobacter freundii* JCM 1657) | 98,26 | MZ934704 |
|  | *Escherichia coli* DH5alpha BRL | 2,29 | *Escherichia coli* ATCC 35218 CHB | 2,33 | *Escherichia coli* NBRC 102203 | 99,64 | MZ934706 |
|  | *Enterobacter cloacae* 13159_1 CHB | 2,10 | *Enterobacter cloacae* DSM 46348 DSM | 2,28 | *Enterobacter hormaechei subs xiangfangensis* 10-17 | 99,79 | MZ934705 |
|  | *Klebsiella pneumoniae ssp pneumoniae* 9295_1 CHB | 2,16 | *Klebsiella pneumoniae ssp pneumoniae* 9295_1 CHB | 2,43 | *Klebsiella pneumoniae* DSM 30104 | 99,64 | MZ934707 |
| DFI-22 | *Candida albicans* ATCC 10231 THL | 1,88 | *Streptomyces badius* B192 UFL | 1,45 | - |  |  |
|  | *Candida krusei* ATCC 6258 VML | 1,92 | *Mycobacterium celatum* DSM 44243T DSM | 1,22 | - |  |  |
|  | *Proteus vulgaris* DSM 13387NT HAM | 2,23 | *Proteus vulgaris* DSM 13625 DSM | 2,37 | *Proteus vulgaris* ATCC 29905 | 99,35 | MZ951127 |
|  | *Staphylococcus aureus ssp aureus* DSM 20232 DSM | 2,06 | *Staphylococcus aureus* ATCC 33591 THL | 2,47 | *Staphylococcus aureus* NBRC 100910 / ATCC 12600 | 100 | MZ951129 |
|  | *Escherichia coli* DH5alpha BRL | 2,00 | *Escherichia coli* DH5alpha BRL | 2,31 | *Escherichia coli* NBRC 102203 | 99,15 | MZ951131 |
|  | *Streptococcus agalactiae* 04_158 CTL | 2,12 | *Streptococcus agalactiae* V29 CTL | 2,47 | *Streptococcus agalactiae* ATCC 13813 | 99,93 | MZ951130 |
|  | *Myroides odoratimimus* LMG 4029T HAM | 1,90 | Myroides odoratimimus LMG 4029T HAM | 2,27 | *Myroides odoratimimus* CCUG 39352 | 99,85 | MZ951134 |
|  | *Enterococcus faecalis* | 2,20 | *Enterococcus faecalis* DSM 2570 DSM | 2,34 | *Enterococcus faecalis* ATCC 19433 | 100 | MZ951133 |
|  | *Staphylococcus epidermidis* CCM 4505 CCM | 1,87 | *Staphylococcus epidermidis* ATCC 14990T THL | 1,91 | *Staphylococcus epidermidis* NBRC 100911 | 100 | MZ951138 |
|  | *Citrobacter freundii* 13158_2 CHB | 1,61 | *Citrobacter freundii* 22054_1 CHB | 2,07 | *Citrobacter freundii* ATCC 8090 = mtcc 1658 | 99,79 | MZ951142 |
| DFI-23 | *Enterococcus faecalis* ATCC 29212 CHB | 2,23 | *Enterococcus faecalis* DSM 6134 DSM | 2,43 | *Enterococcus faecalis* ATCC 19433 | 100 | MZ956264 |
|  | *Staphylococcus simulans* DSM 20323 DSM | 1,96 | *Staphylococcus simulans* DSM 20323 DSM | 2,19 | *Staphylococcus simulans* MK 148 | 99,86 | MZ951146 |
|  | *Escherichia coli* W3350 MMG | 2,06 | *Escherichia coli* DH5alpha BRL | 2,20 | *Escherichia coli* NBRC 102203 | 99,42 | MZ951157 |
|  | *Staphylococcus aureus* ATCC 33591 THL | 1,91 | *Staphylococcus aureus* ATCC 33591 THL | 2,38 | *Staphylococcus aureus* NRBC 100910 | 100 | MZ951147 |
|  | *Streptococcus pyogenes* ATCC 19615 THL | 2,10 | *Streptococcus pyogenes* ATCC 19615 THL | 2,36 | *Streptococcus pyogenes* JCM 5674 | 100 | MZ951159 |
| DFI-24 | *Enterococcus faecalis* ATCC 7080 THL | 2,24 | *Enterococcus faecalis* DSM 20409 DSM | 2,44 | *Enterococcus faecalis* ATCC 19433 | 100 | MZ951163 |
|  | *Escherichia coli* DH5alpha BRL | 2,16 | *Escherichia coli* DH5alpha BRL | 2,51 | *Escherichia coli* NRBC 102203 | 99,34 | MZ951164 |
|  | *Enterobacter cloacae* MB_8779_05 THL | 2,05 | *Enterobacter cloacae* MB_8779_05 THL | 2,18 | *Enterobacter cloacae* ATCC 13047 | 99,78 | MZ951167 |
|  | *Proteus mirabilis* 9482_2 CHB | 2,08 | *Proteus mirabilis* DSM 46227 DSM | 2,34 | *Proteus mirabilis* ATCC 29906 | 99,93 | MZ951168 |
|  | *Staphylococcus simulans* DSM 20723 DSM | 1,62 | *Staphylococcus simulans* DSM 20723 DSM | 1,93 | *Staphylococcus simulans* MK 148 | 99,86 | MZ951169 |
| DFI-25 | *Sphingobacterium multivorum* DSM 11691T HAM | 2,39 | *Sphingobacterium multivorum* DSM 11691T HAM | 2,22 | *Sphingobacterium multivorum* NBRC 14947 | 99,71 | MZ955455 |
|  | *Staphylococcus aureus* ATCC 33591 THL | 2,40 | *Staphylococcus aureus ssp aureus* DSM 3463 DSM | 2,37 | *Staphylococcus aureus* NBRC 100910 | 99,93 | MZ956162 |
|  | *Staphylococcus epidermidis* 10547 CHB | 2,12 | *Staphylococcus epidermidis* 10547 CHB | 2,16 | *Staphylococcus epidermidis Fussel* | 100 | MZ956274 |
| DFI-26 | *Proteus mirabilis* 9482_2 CHB | 2,42 | *Proteus mirabilis* 13210_1 CHB | 2,33 | *Proteus mirabilis* JCM 1669 | 99,93 | MZ955635 |
|  | *Enterococcus faecalis* ATCC 29212 CHB | 2,36 | *Enterococcus faecalis* 20247_4 CHB | 2,46 | *Enterococcus faecalis* ATCC 19433 | 100 | MZ955864 |
|  | *Morganella morganii* (E) 21086317 MLD | 2,48 | *Morganella morganii ssp morganii* 15284_1 CHB | 2,50 | *Morganella morganii* LMG 7874 | 99,85 | MZ955868 |
|  | *Streptococcus agalactiae* V29 CTL | 2,44 | *Streptococcus agalactiae* V29 CTL | 2,51 | *Streptococcus agalactiae* ATCC 13813 | 99,93 | MZ955884 |
| DFI-27 | *Enterococcus faecalis* ATCC 7080 THL | 2,39 | *Enterococcus faecalis* ATCC 7080 THL | 2,42 | *Enterococcus faecalis* ATCC 19433 | 100 | MZ955986 |
|  | *Acinetobacter pittii* DSM 9321 DSM | 2,24 | *Acinetobacter pittii* DSM 9321 DSM | 2,27 | *Acinetobacter pittii* DSM 21653 | 99,79 | MZ955994 |
|  | *Klebsiella pneumoniae ssp pneumoniae* 9295_1 CHB | 2,29 | *Klebsiella pneumoniae ssp pneumoniae* 9295_1 CHB | 2,38 | *Klebsiella pneumoniae* DSM 30104 | 99,50 | MZ956116 |
| DFI-28 | *Enterococcus faecalis* ATCC 29212 CHB | 2,33 | *Enterococcus faecalis* ATCC 7080 THL | 2,51 | *Enterococcus faecalis* ATCC 19433 | 100 | MZ956119 |
|  | *Citrobacter koseri* DSM 4570 DSM | 2,37 | *Citrobacter koseri* 9553_1 CHB | 2,58 | *Citrobacter koseri* LMG 5519 | 99,36 | MZ956120 |
|  | *Morganella morganii* (E) 21086317 MLD | 2,38 | *Morganella morganii* RV_BA_03_A LBK | 2,56 | *Morganella morganii* NBRC 3848 | 99,78 | MZ956128 |
|  | *Escherichia coli* DH5alpha BRL | 2,21 | *Escherichia coli* DH5alpha BRL | 2,43 | *Escherichia coli* NBRC 102203 | 99,20 | MZ956129 |
|  | *Acinetobacter haemolyticus* LMG 1033 HAM | 2,39 | *Acinetobacter junii* DSM 6964T HAM | 2,12 | *Acinetobacter gyllenbergii* RUH 422 | 100 | MZ956131 |
| DFI-29 | *Escherichia coli* MB11464_1 CHB | 2,17 | *Escherichia coli* ATCC 25922 CHB | 2,23 | *Escherichia coli* NBRC 102203 | 99,71 | MZ956132 |
|  | *Corynebacterium striatum* 23086514 MLD | 2,28 | *Corynebacterium striatum* 143 RLT | 2,16 | *Corynebacterium striatum* ATCC 6940 | 99,77 | MZ956136 |
|  | *Enterococcus faecalis* ATCC 7080 THL | 2,02 | *Enterococcus faecalis* 20247_4 CHB | 2,29 | *Enterococcus faecalis* ATCC 19433 | 100 | MZ956134 |
| DFI-30 | *Enterococcus faecalis* 20247_4 CHB | 2,43 | *Enterococcus faecalis* 20247_4 CHB | 2,36 | *Enterococcus faecalis* ATCC 19433 | 100 | MZ960142 |
|  | *Escherichia coli* MB11464_1 CHB | 2,38 | *Escherichia coli* MB11464_1 CHB | 2,35 | *Escherichia coli* NBRC 102203 | 99,57 | MZ960143 |
|  | *Staphylococcus warneri* Mb18796_1 CHB | 1,94 | \| *Staphylococcus warneri* CCM 2604 CCM \| \| --- \| | 1,85 | *Staphylococcus warneri* AW 25 | 99,93 | MZ960144 |
| DFI-31 | \| *Streptococcus agalactiae* 04_158 CTL \| \| --- \| | 2,39 | \| *Streptococcus agalactiae* 04_158 CTL \| \| --- \| | 2,41 | *Streptococcus agalactiae* ATCC 13813 | 99,93 | MZ960149 |
|  | \| *Escherichia coli* DH5alpha BRL \| \| --- \| | 2,29 | *Escherichia coli* DH5alpha BRL | 2,29 | *Shigella dysenteriae* ATCC 13313 | 99,78 | MZ960146 |
|  | *Enterococcus faecalis* ATCC 7080 THL | 2,34 | *Enterococcus faecalis* ATCC 7080 THL | 2,26 | *Enterococcus faecalis* ATCC 19433 | 100 | MZ960150 |

* National Center for Biotechnology Information

- lost during passaging

? failed PCR (non-specific products)

**Genus confidence – 98%**

**Species confidence – 93%**

**Supplemenatary Table S2.**

| Temperature program | | | | | Set |
| --- | --- | --- | --- | --- | --- |
|  | 30 cycles | | |  | 1, 2 |
| 94°C | 94°C | 60°C | 72°C | 72°C |  |
| 10 min | 40 s | 40 s | 60 s | 7 min |  |
|  | 36 cycles | | |  | 3-6 |
| 94°C | 94°C | 52°C | 72°C | 72°C |  |
| 10 min | 30 s | 40 s | 50 s | 5 min |  |
|  | 30 cycles | | |  | 7 |
| 95°C | 94°C | 57°C | 72°C | 72°C |  |
| 15 min | 30 s | 90 s | 90 s | 10 min |  |
|  | 30 cycles | | |  | 8 |
| 94°C | 94°C | 60°C | 72°C | 72°C |  |
| 3 min | 30 s | 30 s | 60 s | 7 min |  |
